# Characterization of Host Responses during Pseudomonas aeruginosa Acute Infection in the Lungs and Blood and after Treatment with the Synthetic Immunomodulatory Peptide IDR-1002

**DOI:** 10.1128/IAI.00661-18

**Published:** 2018-12-19

**Authors:** Kelli Wuerth, Amy H. Y. Lee, Reza Falsafi, Erin E. Gill, Robert E. W. Hancock

**Affiliations:** a Centre for Microbial Diseases and Immunity Research, Department of Microbiology and Immunology, University of British Columbia, Vancouver, British Columbia, Canada; New York University School of Medicine

**Keywords:** *Pseudomonas aeruginosa*, bioinformatics, host-pathogen interactions, immunology, lung infection

## Abstract

Pseudomonas aeruginosa is an opportunistic pathogen that causes nosocomial pneumonia and infects patients with cystic fibrosis. P. aeruginosa lung infections are difficult to treat due to bacterial resistance to antibiotics, and strains with multidrug resistance are becoming more prevalent. Here, we examined the use of a small host defense peptide, innate defense regulator 1002 (IDR-1002), in an acute P. aeruginosa lung infection *in vivo*. IDR-1002 significantly reduced the bacterial burden in bronchoalveolar lavage fluid (BALF), as well as MCP-1 in BALF and serum, KC in serum, and interleukin 6 (IL-6) in BALF. Transcriptome sequencing (RNA-Seq) was conducted on lungs and whole blood, and the effects of P. aeruginosa, IDR-1002, and the combination of P. aeruginosa and IDR-1002 were evaluated. Differential gene expression analysis showed that P. aeruginosa increased multiple inflammatory and innate immune pathways, as well as affected hemostasis, matrix metalloproteinases, collagen biosynthesis, and various metabolism pathways in the lungs and/or blood. Infected mice treated with IDR-1002 had significant changes in gene expression compared to untreated infected mice, with fewer differentially expressed genes associated with the inflammatory and innate immune responses to microbial infection, and treatment also affected morphogenesis, certain metabolic pathways, and lymphocyte activation. Overall, these results showed that IDR-1002 was effective in treating P. aeruginosa acute lung infections and associated inflammation.

## Abstract (precis)

Pseudomonas aeruginosa is an opportunistic pathogen that causes nosocomial pneumonia and infects patients with cystic fibrosis. P. aeruginosa lung infections are difficult to treat due to bacterial resistance to antibiotics, and strains with multidrug resistance are becoming more prevalent.

## INTRODUCTION

Pseudomonas aeruginosa is a common source of infections caused by medical devices, such as catheters, and also frequently infects burns and wounds (1, 2). However, its roles in lung infections are among the most concerning. P. aeruginosa is one of the leading causes of ventilator-associated pneumonia and hospital-acquired pneumonia, particularly in the intensive care unit or in late-onset cases (3–5). P. aeruginosa has also been found in patients with chronic obstructive pulmonary disease (COPD) (6). Critically, it chronically infects the lungs of patients with cystic fibrosis (CF), with the majority of patients becoming infected by their mid-20s, and P. aeruginosa is associated with increased hospitalization and mortality in CF patients (7, 8). Treatment of P. aeruginosa lung infections is difficult due to the organism’s inherent, adaptive, and acquired antibiotic resistance mechanisms, as well as challenges in delivering drugs to the lung environment (9, 10). Multidrug resistance in P. aeruginosa is increasing, necessitating a search for new treatment options (11).

Host defense peptides (HDPs), also called antimicrobial peptides (AMPs), are small amphipathic peptides (10 to 50 amino acids) that are typically cationic (charge, +2 to +9). They demonstrate numerous anti-inflammatory and anti-infective effects against microbial infections, but previous work using HDPs against *in vivo* P. aeruginosa respiratory tract infections has had only limited success (12–18). Many of the peptides demonstrated toxicity *in vivo*, or their effects on inflammation, a key feature of P. aeruginosa lung infections, were not fully examined (12–18). None of these peptides has advanced to clinical trials for the treatment of P. aeruginosa infections. Recently, we showed that one HDP, innate defense regulator 1002 (IDR-1002), was effective against the inflammatory sequelae of P. aeruginosa infections in a model using the polysaccharide alginate to mimic a chronic infection without showing toxicity (19). Here, the aim was to explore the mechanisms underlying IDR-1002 activities in an acute P. aeruginosa lung infection model, as well as to examine the effects of the P. aeruginosa infection itself using systems biology methods.

IDR-1002 (VQRWLIVWRIRK-NH2) is a synthetic derivative of the bovine HDP bactenecin (RLCRIVVIRVCR-NH2), with amino acid substitutions used to create a linearized peptide that shows improved immunomodulatory activity compared to bactenecin (20, 21). Previous work on IDR-1002 in a Staphylococcus aureus intraperitoneal (i.p.) infection model demonstrated that it helped to recruit leukocytes to the infection site, with an increase seen in both neutrophils and the neutrophil chemokine KC (20). Increased numbers of monocytes were also observed, although no changes in monocyte chemoattractant protein 1 (MCP-1) expression were seen (20). Eliminating macrophages with liposomal clodronate removed the protective effect of IDR-1002 (20). This indicates that a key factor in IDR-1002-mediated protection in the S. aureus intraperitoneal model was the recruitment of macrophages, although there are some reports indicating that liposomal clodronate also depletes dendritic cells (DCs) (22, 23). Similar results were achieved with another peptide, IDR-1, against S. aureus infection, with macrophages and monocytes required for protection, while it was additionally shown that depleting neutrophils, T cells, or B cells had no effect (24). In vitro studies with human monocytes showed that IDR-1002 can promote cell adhesion to fibronectin in the presence of chemokines due to its increased activation of β-integrins and the phosphatidylinositol 3-kinase (PI3K)-Akt pathway, and the peptide also increased the expression of the chemokine receptor CCR5 (25, 26). IDR-1002 also reduced inflammation in a sterile ear inflammation model, which was attributed to its repression of class A/1 rhodopsin-like G protein-coupled receptors, the interferon gamma (IFN-γ) response, and regulation by IRF8 (27). Therefore, while some aspects of IDR-1002 mechanisms have been uncovered and indicate its involvement in leukocyte recruitment, IDR-1002 has not been thoroughly examined in the context of infections, especially lung infections by P. aeruginosa. Additionally, we decided to take a more comprehensive approach to the evaluation of its mechanisms of action. Therefore, transcriptome sequencing (RNA-Seq) was utilized in conjunction with advanced bioinformatics appraisal of the host immune response, both locally in the lungs and systemically in the blood. RNA-Seq is a powerful method for evaluating the transcriptome of an organism. It uses sequencing by synthesis and does not require the use of probes, as with microarray technology, thereby allowing more efficient and accurate discovery of dysregulated transcripts without substantial and variable backgrounds, as seen for hybridization methods, such as microarrays (28).

While the host response to murine P. aeruginosa lung infections has been evaluated using microarrays (29, 30), the use of RNA-Seq has been limited. To our knowledge, only one study has used (dual) RNA-Seq for a P. aeruginosa lung infection model, and it examined only the response in the lungs and utilized a weakly virulent isolate, PAO1, at very high input doses (2 × 10^8^ CFU) to define only 702 host genes changing expression (31). In contrast, here, we performed RNA-Seq on both the lungs and blood from infected mice after infection with lower input doses (∼8 × 10^5^ CFU) of a highly virulent isolate strain, PA103, to provide new insights into the effects of acute, rapidly progressing P. aeruginosa infections and possibly uncover new drug targets.

The RNA-Seq results showed that P. aeruginosa caused >4,700 genes to change expression in the lungs, with profound inflammatory and immune responses in both the lungs and the blood, and also demonstrated the involvement of novel biological processes. While the mice given IDR-1002 alone showed few changes in gene expression compared to the negative-control group, differences in hemostasis and other processes among the infected mice after IDR-1002 treatment of PA103-infected mice provide new leads for understanding IDR mechanisms of action. Critically, in these experiments, IDR-1002 led to reductions in the CFU burden, the inflammatory cytokine and chemokine levels, and the associated inflammatory pathways.

## RESULTS

### IDR-1002 reduced P. aeruginosa burden and inflammation in the lungs and did not itself produce inflammatory cytokines.

To examine the effects of IDR-1002, P. aeruginosa, or their combination, an acute P. aeruginosa lung model was used. Female C57BL/6J mice 6 to 8 weeks of age were given 8 mg/kg of body weight IDR-1002 or the vehicle (endotoxin-free water) intranasally (i.n.) 24 h prior to infection with ∼8 × 10^5^ CFU/mouse of the virulent P. aeruginosa strain PA103 or the vehicle (endotoxin-free saline) and then euthanized, and all samples were collected at 18 h postinfection. Prophylactic treatment was used to eliminate any direct antimicrobial effects of IDR-1002 on the bacteria, since our goal was to focus on characterizing the immunomodulatory activities of IDR-1002. The groups of mice are referred to here as negative-control (which received only vehicle), IDR-1002 control (which received IDR-1002 and saline), PA103-infected (which received water and PA103), and IDR-1002 treatment (which received IDR-1002 and PA103) mice.

**FIG 1:**
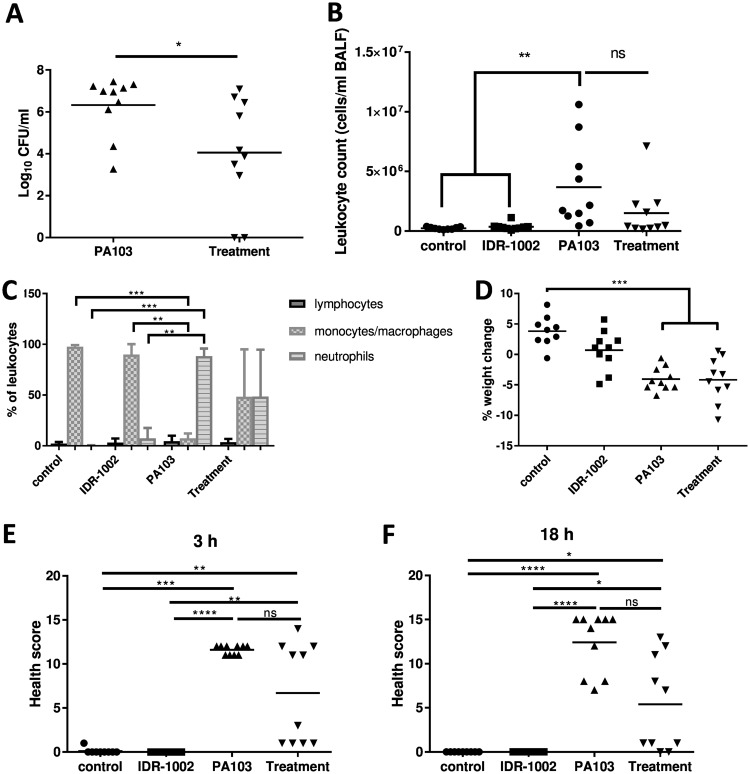
IDR-1002 treatment reduced the CFU burden in mice infected with P. aeruginosa PA103. Mice were given water or IDR-1002 at 24 h prior to infection (−24 h), given saline or P. aeruginosa PA103 at 0 h, and then euthanized, and samples were processed at 18 h. (A) CFU counts from BALF. (B) Leukocyte counts in BALF. (C) Distribution of leukocytes in BALF, represented as means and standard errors of the mean (SEM). (D) Percent weight loss. (E and F) Health scores at 3 h or 18 h postinfection. The data represent 9 or 10 mice per condition from a combination of two experiments and were analyzed using an unpaired two-tailed *t* test for the CFU (A), a one-way ANOVA and Tukey’s multiple-comparison test for the total leukocytes (B), and a Kruskal-Wallis test with Dunn’s multiple-comparison test (C to F). *, *P* ≤ 0.05; **, *P* ≤ 0.01; ***, *P* ≤ 0.001; ****, *P* ≤ 0.0001; ns, not significant.

Prophylactic treatment with IDR-1002 significantly decreased the PA103 CFU burden in the lungs more than 100-fold compared to that in PA103-infected mice (Fig. 1). Infection with PA103 significantly increased leukocyte infiltration into the lungs compared to either the negative-control or IDR-1002 control mice, whereas the IDR-1002 treatment mice showed a leukocyte count that was lower than that of the PA103-infected mice but higher than that of the negative-control or IDR-1002 control mice, although none of the comparisons involving IDR-1002 treatment mice was significant. The increase in the total leukocyte count of IDR-1002 treatment mice was not significant compared to negative-control or IDR-1002 control mice (Fig. 1B). As expected, the PA103-infected mice showed a very strong increase in neutrophils compared to the uninfected control mice (Fig. 1C), while monocytes became a relatively minor proportion of leukocytes. In contrast, the treatment mice showed similar proportions of neutrophils and monocytes/macrophages that favored neutrophils in sicker mice, while mice that had few signs of infection showed more monocytes/macrophages. Both the PA103 and treatment groups had slight but significant weight loss compared to the negative-control mice (Fig. 1D), and although the health scores showed improvement in the IDR-1002 treatment group at both 3 and 18 h postinfection compared to the PA103-infected mice, these differences were not significant (Fig. 1E and F).

**FIG 2:**
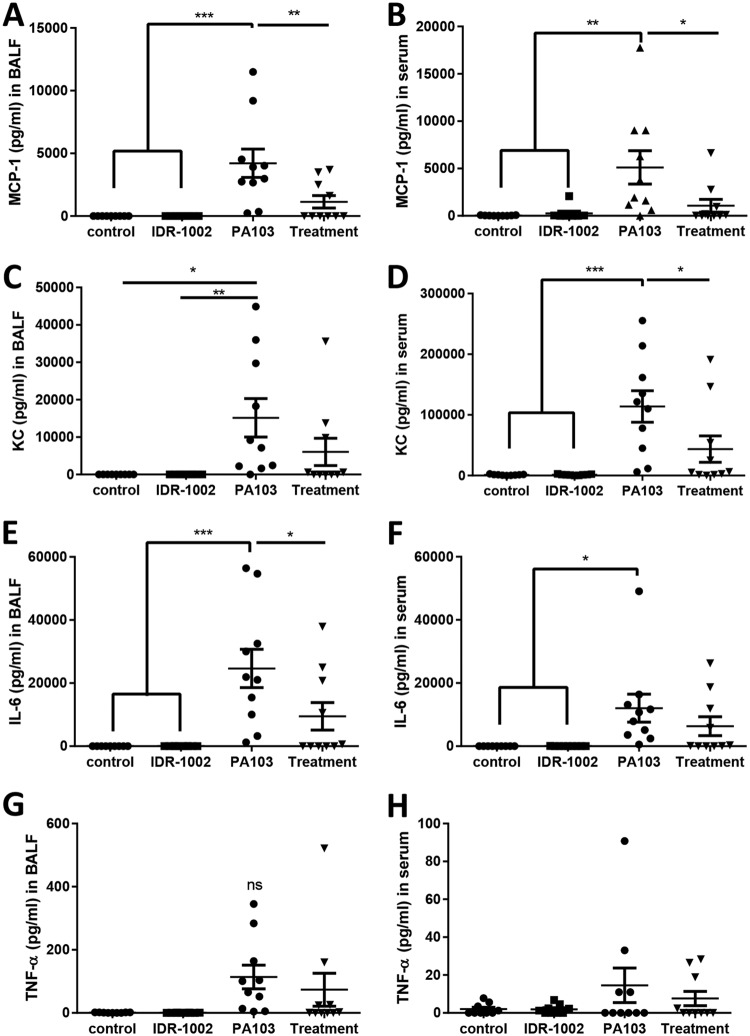
IDR-1002 treatment reduced cytokines and chemokines in BALF and serum that were induced by P. aeruginosa PA103. Mice were given water or IDR-1002 at −24 h, given saline or P. aeruginosa PA103 at 0 h, and then euthanized, and samples were processed at 18 h. ELISAs were performed for MCP-1 in BALF (A) and serum (B), KC in BALF (C) and serum (D), IL-6 in BALF (E) and serum (F), and TNF-α in BALF (G) and serum (H). The data represent mean ± SEM for 9 or 10 mice per condition from a combination of two experiments and were analyzed using one-way ANOVA and Tukey’s multiple-comparison test. *, *P* ≤ 0.05; **, *P* ≤ 0.01; ***, *P* ≤ 0.001; ns, not significant.

The expression of cytokines and chemokines in the bronchoalveolar lavage fluid (BALF) and serum was also examined (Fig. 2). MCP-1, KC, and interleukin 6 (IL-6) showed significant increases in the BALF and sera of the PA103-infected mice compared to either negative-control or IDR-1002 control mice, while the IDR-1002 treatment mice had significant decreases in most of these outputs compared to the PA103 mice. Tumor necrosis factor alpha (TNF-α) also showed similar trends. Notably, there were no significant increases in any of the tested cytokines or chemokines for IDR-1002 control compared to negative-control mice.

### P. aeruginosa lung infection induced profound changes in the transcriptome of both the lungs and blood.

The lungs and whole blood from one of two experiments, consisting of 4 or 5 mice per condition, were used to isolate RNA and to run RNA-Seq to characterize the differentially expressed (DE) genes of the host transcriptome during infection and treatment with IDR-1002. The total numbers of host DE genes for several comparisons are shown in Table 1. Infection with PA103 led to the differential expression of 4,739 genes in the lungs and 1,327 in the blood compared to the negative-control samples. In the lungs, there were 2,360 upregulated DE genes and 2,379 downregulated DE genes after PA103 infection compared to the negative-control mice. The upregulated DE genes in the PA103-infected lungs comprised multiple genes encoding innate immune and inflammatory response proteins, including the mouse cathelicidin CRAMP, acute-phase serum amyloid A proteins, and numerous chemokines. There were also genes for several matrix metalloproteinases (MMPs) that were upregulated (MMP-3, -8, -9, -12, -14, and -25). The genes for the cytokines and chemokines examined in the enzyme-linked immunosorbent assays (ELISAs), IL-6, TNF-α, MCP-1, and KC, were all upregulated in the PA103-infected mice, similar to the results seen at the protein level in Fig. 2, with these genes showing greater-than-32-fold changes (FC) compared to the control mice. The downregulated DE genes in the lungs were more varied in function but, notably, included several genes encoding subunits of various types of collagen, which is a major component of the extracellular matrix (ECM) (32).

**TABLE 1:** Numbers of host DE genes for different comparisons in the lungs and blood

Comparison	No. of genes	
	Lungs	Blood
IDR-1002 control vs. negative control	2	0
PA103 infected vs. negative control	4,739	1,327
IDR-1002 treatment vs. negative control	813	294
IDR-1002 treatment vs. IDR-1002 control	638	271
IDR-1002 treatment vs. PA103 infected	2,111	1

While the top DE genes according to fold change provide some interesting insights, it is important to take a systemic approach to the data. Therefore, overrepresented pathways were discerned among DE genes using signature overrepresentation analysis (SIGORA), a gene pair overrepresentation analysis tool that is designed to reveal specific processes that are relevant to the model under consideration (33). Unlike many other pathway tools, SIGORA limits repetition of pathways (i.e., reduces the identification of certain pathways due to genes annotated to multiple pathways) by performing gene pair-based pathway enrichment. For PA103-infected versus negative-control mice, 59 pathways were enriched among the DE genes (Table 2). The top two pathways identified were hemostasis and axon guidance, which have not been identified in most previous infection studies. Hemostasis is often associated with sepsis and can be targeted by inflammatory mediators (34). While axon guidance is associated with the nervous system, the proteins in the pathway are also involved in the development of other tissues, including the lungs and blood vessels, and have roles in cell migration (35). Among other highly dysregulated pathways, many were involved in inflammation and innate immune responses, including chemokine receptor binding, interferon gamma signaling, Toll-like receptor 5 (TLR5) and MyD88-independent TLR3/4 cascades, and interleukin 1 and other cytokine signaling (Table 2). These results were in agreement with the strong immune response expected as a consequence of an infection, and the roles of both MyD88-dependent and MyD88-independent pathways have been noted in other P. aeruginosa infection models (36, 37).

**TABLE 2:**
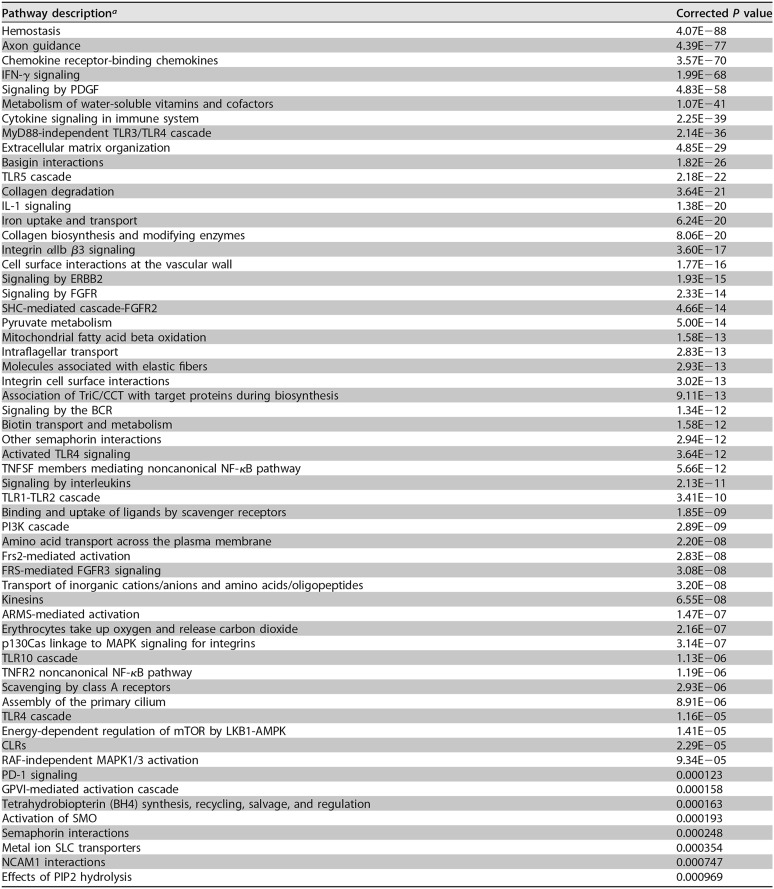
Host pathways dysregulated in the lungs of PA103-infected versus negative-control mice

^a^ PDGF, platelet-derived growth factor; FGFR, fibroblast growth factor receptor; BCR, B cell receptor; TNFSF, TNF receptor superfamily; MAPK, mitogen-activated protein kinase; CLRs, C-type lectin receptors.

**FIG 3:**
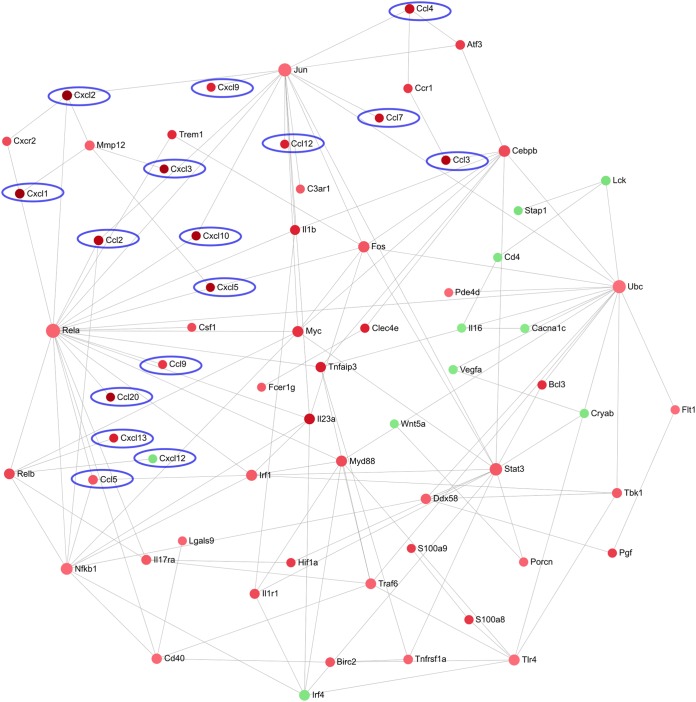
Zero-order protein-protein interaction network of DE genes that are associated with leukocyte migration and their interacting partners in the lungs of PA103-infected versus negative-control mice. The red nodes were upregulated and the green nodes were downregulated in the PA103 group. A darker color indicates a stronger change. Chemokines are circled in blue.

To gain further insights into the genes and molecular interactions involved in some of these pathways, NetworkAnalyst was used to create and visualize protein-protein interaction networks (38). Since the genes for multiple chemokines were upregulated and the chemokine receptor-binding chemokine pathway was dysregulated (Table 2), a zero-order network was created showing the interactions of DE genes associated with leukocyte migration (Fig. 3). Almost all of the genes in the network were upregulated (Fig. 3, red nodes) in the PA103-infected mice compared to the negative-control mice, and numerous chemokines from both the CXC and CC families were observed and interconnected with various transcription factors, including three NF-κB subunits, Jun, and Fos. In agreement with these observations, P. aeruginosa lung infections are known to lead to an influx of leukocytes, particularly neutrophils (39, 40), as confirmed here by an increase in neutrophils in BALF (Fig. 1).

**FIG 4:**
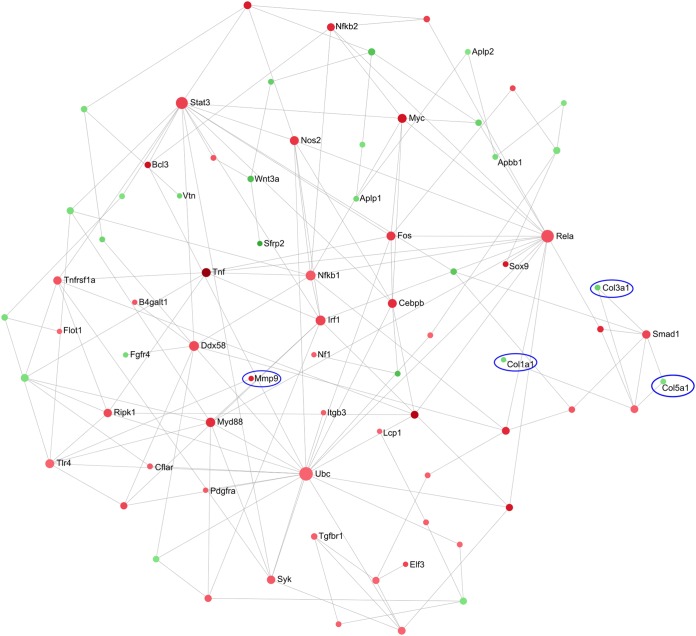
Zero-order protein-protein interaction network of DE genes that are associated with ECM organization and their interacting partners in the lungs of PA103-infected versus negative-control mice. The red nodes were upregulated and the green nodes were downregulated in the PA103 group. A darker color indicates a stronger change. The genes for matrix metalloproteinase MMP-9 and components of collagen are circled in blue.

The pathway analysis also showed multiple pathways related to cell and tissue differentiation or structure, including collagen, integrins, and ECM organization (Table 2). Therefore, genes involved in ECM organization were also used to create a protein-protein interaction network (Fig. 4). The upregulated DE genes included *Mmp9*, which encodes a matrix metalloproteinase that is upregulated and released during numerous lung injury models and that decreases ECM integrity (41). Downregulated DE genes included several for chains of types of collagen, the major fibrous component of the ECM (32). These data are consistent with the breakdown of tissues in the lung.

The RNA-Seq results for the lungs also pointed to substantial changes in metabolism or nutrient acquisition, including metabolism of water-soluble vitamins and cofactors, iron uptake and transport (31), pyruvate metabolism, and mitochondrial fatty acid beta-oxidation (Table 2). Many of these pathways involve genes that were downregulated in the PA103-infected mice compared to the control mice.

In the blood, there were 686 upregulated and 641 downregulated DE genes in the PA103-infected mice compared to the negative-control mice. As with the lungs, several of the most upregulated DE genes in the blood were associated with infection and inflammation, such as *Lcn2*, *Cd177*, *Ngp*, and other genes associated with neutrophil-induced inflammation, and the genes for the host defense peptide CRAMP and iron-sequestering lactotransferrin. There were 729 genes that were differentially expressed in the blood, but not the lungs, which included *Cd72*, *Tnfrsf3*, and complement-associated genes, such as *C1qa* and *C4b*. These differences between the local (lung) and distant (blood) gene expression responses were reflected in the dysregulated pathways (Table 3), with fewer inflammatory pathways involved and the observation of novel pathways. While IL-6, MCP-1, and KC were significantly upregulated in the serum and TNF-α showed a trend toward upregulation, only the genes for MCP-1 and TNF-α were significantly upregulated in the blood, probably reflecting differences in kinetics for these cytokines and chemokines and/or mobilization of some cytokines from the lung into the blood. Among the downregulated DE genes, there were several involved with B cell signaling responses, activation, and antigen presentation. Similarly, SIGORA also demonstrated pathways related to an immune response, including TLR, interferon, and chemokine signaling, as well as major histocompatibility complex (MHC) class II antigen presentation and B cell signaling pathways (Table 3).

**TABLE 3:**
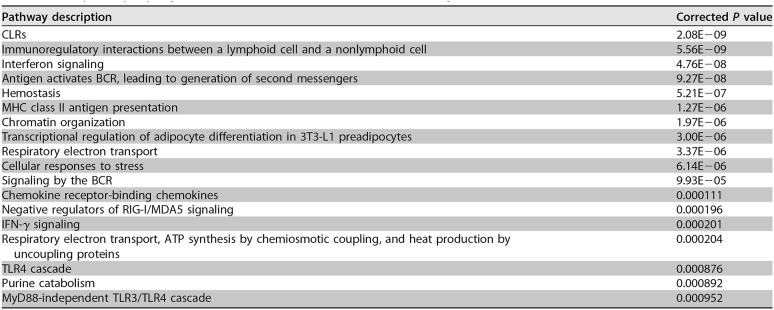
Host pathways dysregulated in the blood of PA103-infected versus negative-control mice

**FIG 5:**
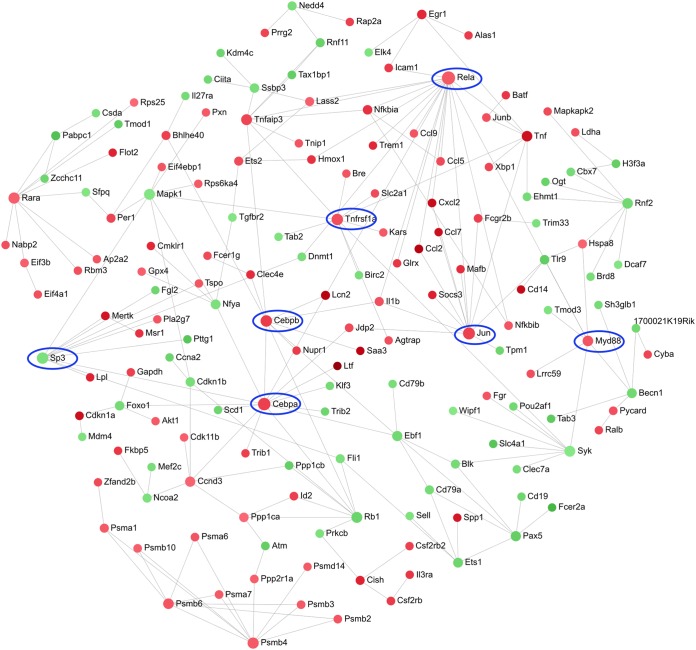
Zero-order protein-protein interaction network of DE genes in the blood of PA103-infected versus negative-control mice. The red nodes were upregulated and the green nodes were downregulated in the PA103 group. A darker color indicates a stronger change. Several key genes involved in the immune response to infection are circled in blue.

A zero-order interaction network for the blood (Fig. 5) showed a mixture of up- and downregulated genes. Prominent hubs for upregulated genes included *Rela*, encoding a subunit of the transcription factor NF-κB; *MyD88*; and *Cebpa* and *Cebpb*, encoding two members of the CCAAT/enhancer binding protein (C/EBP) transcription factor family that are involved in lung inflammation (42). The downregulated gene *Sp3*, which encodes a proteinase inhibitor, was also a hub.

### Administration of IDR-1002 reduced innate immune and inflammatory responses induced by P. aeruginosa in the lungs and blood.

**FIG 6:**
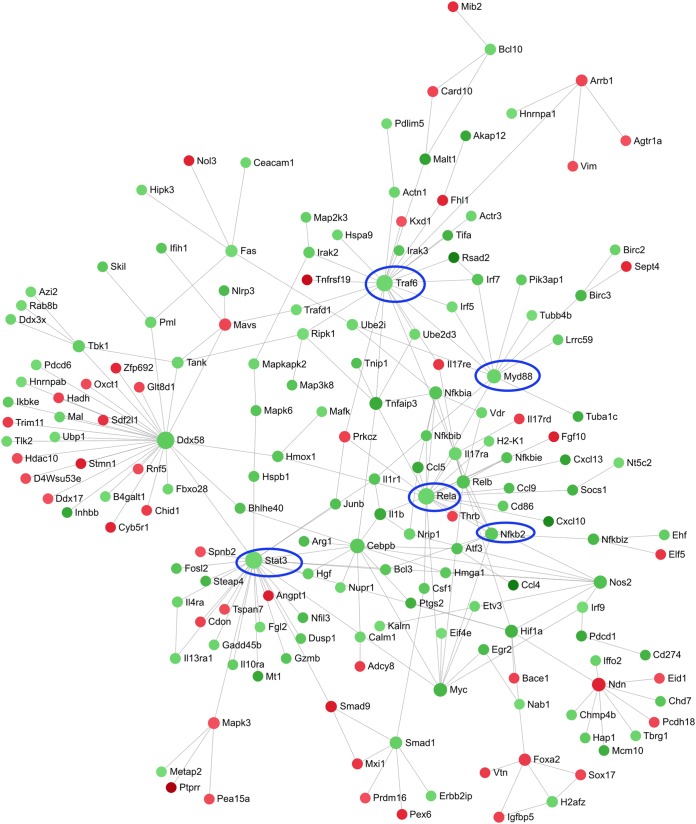
Zero-order protein-protein interaction network of DE genes in the lungs of IDR-1002 treatment versus PA103-infected mice. The red nodes were upregulated and the green nodes were downregulated in the treatment group. A darker color indicates a stronger change. Several key genes involved in the immune response to infection are circled in blue.

The application of IDR-1002 to uninfected mice led to only two DE genes in the lungs, *Csf3* and *Saa2*, the genes encoding granulocyte colony-stimulating factor (G-CSF) and serum amyloid A2, which were upregulated and downregulated, respectively, compared to the negative-control mice. However, while the response in uninfected mice was limited, the prophylactic administration of IDR-1002 in combination with PA103 infection had a large impact on the transcriptome. The comparison between the IDR-1002 treatment and PA103-infected groups showed 2,111 DE genes in the lungs, with 1,110 upregulated and 1,001 downregulated DE genes. The genes for the four proteins examined in ELISAs, namely, MCP-1, KC, IL-6, and TNF-α, showed a trend toward downregulation in the IDR-1002 treatment mice but were not significantly differentially expressed. Compared to the PA103-infected mice, the IDR-1002 treatment mice had the genes for several chemokines downregulated, including *Ccl4*, *Cxcl10*, *Ccl11*, and *Cxcl13*, along with many other inflammatory-response genes. In contrast, the upregulated genes were more diverse. In the overrepresentation analysis, 20 pathways were dysregulated in the IDR-1002 treatment mice compared to PA103-infected mice (Table 4). Four of these pathways, prolonged ERK activation events, IRF3-mediated induction of type I IFN, CRMPs in Sema3A signaling, and hyaluronan uptake and degradation, were not observed in the PA103-infected versus negative control comparison, while the other 16 pathways were also found in this comparison. The pathways common to these two comparisons indicated that the IDR-1002 treatment mice had similarities to the negative-control mice and therefore showed that the IDR-1002 treatment mice generally mitigated (reduced) the effects of infection. Indeed, a zero-order interaction network (Fig. 6) demonstrated that a large number of the downregulated genes in the IDR-1002 treatment mice compared to PA103-infected mice were associated with the inflammatory response, including *Myd88*, *Traf6*, *Rela*, *Nfkb2*, and many chemokine genes. Overall, these results indicated that, while the IDR-1002-treated mice still showed an immune response to infection, the response was muted compared to that of the PA103-infected mice.

**TABLE 4:**
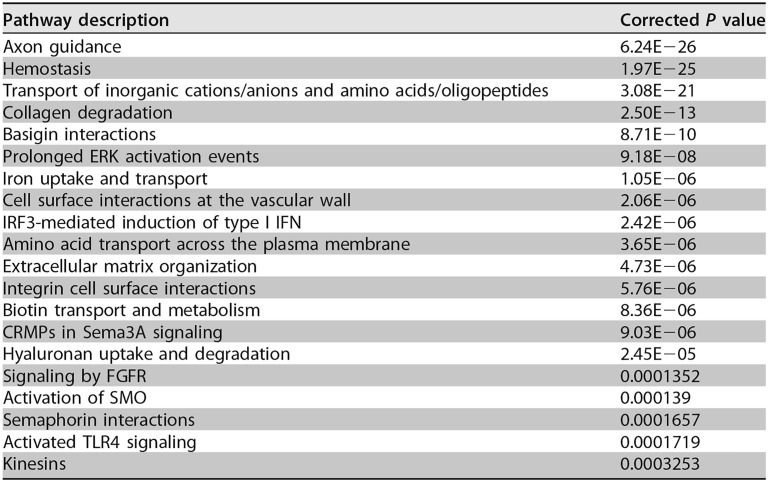
Host pathways dysregulated in the lungs of IDR-1002 treatment versus PA103-infected mice

In the blood, no DE genes were detected for IDR-1002 control mice compared to negative-control mice. There was also only one DE gene related to IDR-1002 treatment versus PA103-infected mice in the blood: *Ighd*, a gene encoding a heavy constant region of IgD, which was upregulated in the treatment group.

## DISCUSSION

Given our interest in characterizing the host immunomodulatory activities of IDR-1002, RNA-Seq analysis was performed on mice with prophylactic treatment with IDR-1002 prior to P. aeruginosa infection to eliminate any potential direct antimicrobial effects of the peptide on the bacteria. Infection with P. aeruginosa PA103 alone led to 4,739 DE host genes in the lungs and 1,327 DE host genes in the blood compared to the uninfected negative-control group. These numbers were greatly reduced by treatment with IDR-1002 in both the lungs and the blood compared to PA103 infection alone, indicating greater similarity of treatment mice with the uninfected negative-control mice. This was in agreement with the reduction in the bacterial CFU burden and the ELISA results indicating reduced cytokine and chemokine expression in the IDR-1002 treatment mice compared to the PA103-infected mice; however, levels remained above those seen in the negative-control or IDR-1002 control mice. The prophylactic use of IDR-1002 to treat PA103 infections led to a range of results with regard to individual mice, with some mice showing either complete or partial elimination of bacterial CFU and signs of inflammation, while a few showed results similar to those of the untreated PA103-infected mice. It is possible that this diversity might reflect the heterogeneity implicit in biological systems in response to both bacteria and peptide, microheterogeneity in the diet or microbiota of mice, or perhaps minor differences in bacterial infectious doses. Indeed, such variation in response to the immune modulator IDR-1002 has been observed with IDRs in other *in vivo* models, including IDR-1018 in a malaria model and IDR-1002 in i.p.-administered Escherichia coli and S. aureus models (20, 43). Increasing the IDR-1002 dose or formulating it to improve delivery might improve its effectiveness in individual mice. Regardless of this heterogeneity, there were still 2,111 DE genes in the lungs of the IDR-1002 treatment compared to those of PA103-infected but untreated mice, with the comparison of IDR-1002 treatment versus PA103-infected mice showing results almost opposite to those of the PA103-infected versus control mice, indicating that the treatment with IDR-1002 reduced the overwhelming inflammatory response induced by PA103, as seen with both the ELISA and the RNA-Seq results. Selected genes identified by RNA-Seq were also validated using quantitative PCR (qPCR) (see Fig. S1 in the supplemental material) and showed trends similar to those in the RNA-Seq results, such as increases in chemokines with PA103 infection that were reduced by treatment with IDR-1002.

There were only two DE genes in IDR-1002 control versus negative-control mice for the lungs and none for the blood. Since the samples were collected 42 h after IDR-1002 administration, it was arguably unsurprising that few changes were seen at the transcriptional level. This indicates that the effect of IDR-1002 is likely to involve priming or polarizing the immune/protective response and that these changes were then potentiated by the additional stimulus of PA103 infection, whereas the mice given only IDR-1002 did not receive an additional signal to the immune system, which then returned essentially to baseline levels. For example, the anti-inflammatory cytokine IL-10 decreases signaling through MHC class II, and IDR-1002 has been shown to increase IL-10 secretion by peritoneal mouse macrophages stimulated *ex vivo*, although the overexpression of IL-10 in P. aeruginosa lung infections could have both beneficial and deleterious effects (20, 44, 45), which might also partly explain the differences in responses in individual IDR-1002 treatment mice.

The RNA-Seq results showed that multiple innate immunity and inflammation pathways were upregulated in the lungs and blood in response to P. aeruginosa. The large number of chemokine genes upregulated was consistent with the increased number of leukocytes seen in the BALF with PA103 infection. Intriguingly, P. aeruginosa lung infection, even in chronic conditions, such as CF, is associated with increased neutrophils in the lungs (40), a feature that was also seen in this acute P. aeruginosa lung infection model, together with the upregulation of many genes associated with neutrophil activation. The upregulation of numerous MMP genes and the decreased expression of genes involved in ECM organization pathways, as observed with the mice infected with PA103, also occurs in the early stages of lung disease, such as CF and COPD (46–49). As these diseases progress, the deposition of certain classes of collagen (particularly I and III) leads to fibrosis and decreases patient respiratory capacity (46, 49).

In conclusion, prophylactic treatment with IDR-1002 reduced bacterial counts and inflammation caused by P. aeruginosa infection. Interestingly, we recently showed that IDR-1002 could also suppress inflammation in an alginate model used to represent chronic lung infection but that it had no effect on bacterial counts in the lung (19). The RNA-Seq results discussed here revealed the differential expression of 4,739 host genes in the lungs (nearly 20% of all of the genes in a mouse) and 1,327 host genes in the blood in response to P. aeruginosa acute lung infection, while the treatment of P. aeruginosa infections with IDR-1002 led to a more muted response than infection alone, with only a few hundred DE genes compared to uninfected controls. Finally, the combination of IDR-1002 and PA103 compared to PA103 alone indicated 2,111 DE genes that were influenced by peptide treatment, in particular revealing a muted innate immune/inflammatory response. These data provided additional insights into P. aeruginosa infection, revealing several new elements not observed previously and possible mechanisms of IDR-1002 protection. Together, these results suggest that IDR-1002 could potentially be used as an adjuvant (perhaps together with antibiotics) to prime the host immune system by modulating the inflammatory response, cytokine production, and immune cell recruitment. Given that IDRs have been shown to have additional activities, such as antibiofilm activity, and demonstrate synergistic effects when used in combination with conventional antibiotics, our future work will focus on identifying the appropriate synergistic combinations to develop IDRs as agents for use against P. aeruginosa acute lung infections (50–52).

## MATERIALS AND METHODS

### Mice and ethics statement.

Female C57BL/6J mice were purchased from Jackson Laboratory or were bred at the Modified Barrier Facility (University of British Columbia [UBC], Vancouver, Canada) and used between 6 and 8 weeks of age. The animals were housed at the Modified Barrier Facility and kept on a standard 12-h/12-h light/dark timed schedule with *ad libitum* access to food and water. All experiments were approved by the UBC Animal Care Committee.

### Reagents.

IDR-1002 (VQRWLIVWRIRK-NH2) was synthesized by Fmoc (9-fluorenylmethoxy carbonyl) chemistry (Kinexus, Vancouver, BC, Canada) and stored at −20°C as a desiccated powder. For experiments, the peptide was resuspended in endotoxin-free water and stored at −20°C.

### Preparation of bacteria and acute *Pseudomonas* lung infection.

The culture was prepared and mice were infected as previously described (19). Briefly, a frozen stock of the bacterial strain P. aeruginosa PA103 was streaked onto LB plates and grown overnight at 37°C; then, individual CFU from the plates were used and were grown overnight in LB at 37°C with shaking. The overnight cultures were diluted 1:50 and grown to an optical density at 600 nm (OD_600_) reading of approximately 0.5. After washing with endotoxin-free 0.9% sodium chloride solution (saline), the cultures were centrifuged, and the supernatant was discarded; then, the bacteria were resuspended in endotoxin-free saline to an OD_600_ of 0.5. The bacteria were then diluted to the final concentration for immediate instillation *in vivo* at ∼8 × 10^5^ CFU/mouse.

Mice were anesthetized with isoflurane (2 to 5%), placed on an intubation stand (BrainTree Scientific, Braintree, MA, USA), and given IDR-1002 (10 to 20 µl, depending on mouse weight) or endotoxin-free water; then, after 24 h, the mice were again anesthetized, placed on the intubation stand, and given either P. aeruginosa (20 µl) or endotoxin-free saline (20 µl). The solutions were instilled dropwise into the left nostril of each mouse using a micropipette, with periodic administration of isoflurane to maintain a steady respiratory rate. After instillation, the mice were kept on the intubation stand under isoflurane for 2 to 3 min to ensure absorption of the liquid. The mice were monitored at 3 and 18 h postinfection and assigned health scores based on a scoring sheet approved by the UBC Animal Care Committee (see Table S1 in the supplemental material).

The mice were euthanized with an intraperitoneal injection of sodium pentobarbital (120 mg/kg). Blood was collected from the inferior vena cava, and 100 µl was placed in RNAprotect animal blood tubes (Qiagen, Hilden, Germany) for RNA isolation according to the manufacturer’s protocol. The remaining blood was allowed to clot and then centrifuged, and the serum was collected and stored at −20°C until it was used for ELISAs. For BALF collection, the chest cavity and trachea were exposed, and an incision was made in the trachea. A cannulated needle was then inserted and used to slowly fill the lungs with sterile phosphate-buffered saline (PBS) (600 µl), which was then slowly withdrawn through the cannulated needle and saved. This procedure was repeated twice for a total of three washes. After the lavage, the smallest lobe of the lung was placed in RNAlater (Qiagen) and saved for RNA-Seq according to the manufacturer’s protocol.

The first BALF wash was used for CFU enumeration by spread plating undiluted BALF or 10-fold dilutions made in PBS onto LB agar plates in duplicate. The plates were incubated overnight at 37°C, and CFU were enumerated the following day. The remaining first BALF wash was centrifuged, and the supernatant was saved at −20°C until it was used for ELISAs. The pellet from the first BALF wash was combined with the pellet from BALF washes 2 and 3 and resuspended in PBS; then, leukocytes were counted on a hemocytometer using Turk’s stain (Ricca Chemical Company).

The leukocytes were also used in a StatSpin Cytofuge 2 (Beckman-Coulter), and the resulting slides were air dried overnight and stained with a Diff-Quik staining kit (VWR, Radnor, PA, USA) according to the manufacturer’s protocol, and then 200 cells/slide were counted.

### ELISAs.

The levels of cytokines and chemokines were measured using antibodies and standards from eBioscience (San Diego, CA, USA) for TNF-α and IL-6, eBioscience or R&D Systems (Minneapolis, MN, USA) for MCP-1, and Fitzgerald (Acton, MA, USA) or R&D Systems for KC. The ELISAs were performed according to the manufacturers’ protocols, with optimization of antibody and sample dilutions, washes, and incubation times. ELISAs were developed with TMB (eBioscience), and the enzymatic reactions were stopped with 2 N sulfuric acid. The plates were read on a Power Wave X340 plate reader (Bio-Tek Instruments, Winooski, VT, USA), and the data were fitted to a 4-parameter standard curve using KC4 software (Bio-Tek).

### RNA isolation and RNA-Seq.

Total RNA was isolated from the lungs and blood from one experiment (*n* = 5 per condition) using an RNeasy Plus Mini kit (Qiagen). The quality of the RNA was analyzed using an RNA 6000 Nano chip (Agilent Technologies, Santa Clara, CA, USA) on an Agilent 2100 Bioanalyzer; all the samples showed excellent quality, with RNA integrity number (RIN) values greater than 8. Enrichment with poly(dT) beads (New England Biolabs, Ipswich, MA, USA) was then used to isolate the mRNA, and a Kapa stranded total RNA-Seq kit (Kapa Biosystems, Wilmington, MA, USA) was used to create the cDNA libraries. In brief, first-strand cDNA was synthesized, followed by second-strand synthesis and blunt-end formation. After 3′ adenylation, adapters (Bio Scientific, Austin, TX, USA) for multiplexing were ligated, followed by amplification and then purification using Agencourt Ampure XP beads (Beckman Coulter). The quality of the library was checked using a high-sensitivity DNA chip (Agilent, Santa Clara, CA, USA) on an Agilent 2100 Bioanalyzer, and all the samples were shown to have optimal fragment size distributions. The libraries were sequenced on an Illumina (San Diego, CA, USA) GAIIx (lungs) or HiSeq 2500 Rapid Run (blood).

After demultiplexing, the resulting FASTQ files were aligned to the Ensembl murine reference genome GRCm38.p5 (build 86) using STAR aligner (version 2.5.2B) (53). Read count tables were generated using HTSeq-count (version 0.6.1p1) (54). DESeq2 (1.14.1) in R (3.3.2) was used for finding DE genes, with cutoffs for DE genes set at a fold change of ±1.5, equivalent to a log_2_ FC of ±0.58, and an adjusted *P* value (false-discovery rate) of <0.05 (55, 56). One mouse in the PA103 group was inadequately infected and was removed from the analysis for lungs and blood. All the remaining lung samples had excellent quality and numbers of read counts. For the blood, samples with <800,000 read counts were excluded, leaving 3 per condition, except for the PA103 group, which had 4. The DE genes were analyzed with SIGORA for pathway enrichment analysis using the Reactome gene annotation system, with an adjusted *P* value cutoff of <0.001, as recommended by the SIGORA manual (33). The DE genes were used in NetworkAnalyst for network visualization using the Imex database (38).

Lung samples were also used for validation in qPCR. The RNA isolated for RNA-Seq was transcribed into cDNA using a cDNA synthesis kit from Quanta Biosciences (Beverly, MA, USA). The cDNA was used for real-time qPCR using two-step SYBR Green qPCR master mix from Roche (Basel, Switzerland) and primers (see Table S2 in the supplemental material) from Thermo Fisher (Waltham, MA, USA). Fold changes were calculated based on the cycle threshold (*C_T_*) value method using the average *C_T_* value of two housekeeping genes, *Eef2* and *B2m*, and values compared to the control group. The cycle threshold (*C_T_*) is defined as the number of cycles required for the fluorescent signal to exceed the background fluorescence.

### Statistical analysis.

Data from the lung model were analyzed using Microsoft Excel 2013 and GraphPad Prism version 7. GraphPad Prism was used to perform an unpaired two-tailed *t* test or one-way analysis of variance (ANOVA) with Tukey’s multiple-comparison tests. A *P* value of ≤0.05 was considered statistically significant.

### Accession number(s).

The RNA transcriptomic data have been submitted to the National Center for Biotechnology Information Gene Expression Omnibus under accession number GSE110415.

## Supplementary Material

Supplemental file 1
