# Supplementary material for: Characterization of Host Responses during Pseudomonas aeruginosa Acute Infection in the Lungs and Blood and after Treatment with the Synthetic Immunomodulatory Peptide IDR-1002
Source: Infect Immun. 2018 Dec 19;87(1):e00661-18. doi: 10.1128/IAI.00661-18 (PMC6300642; doi:10.1128/IAI.00661-18)
Supplement: Supplemental file 1 [file 056a2c1af0966b83936e05d2c87df8e2_IAI.00661-18-s0001.pdf]

Table S1: Primers used for qPCR.

Name	Forward primer (5’-3’)	Reverse Primer (5’-3’)
B2M	CAGAAAACCCCTCAAATTCAAGTAT	AATTCAGTGTGAGCCAGGATATAGA
CCL3	TGCCCTTGCTGTTCTTCTCT	GTGGAATCTTCCGGCTGTAG
CXCL10	CCAAGTGCTGCCGTCATTTTC	GGCTCGCAGGGATGATTTCAA
EEF2	TGTCAGTCATCGCCCATGTG	CATCCTTGCGAGTGTCAGTGA
KC/CXCL1	CTGGGATTCACCTCAAGAACATC	CAGGGTCAAGGCAAGCCTC
TNFRSF19	CCCTCAATCCCGAAAACGA	TGGCCGCCACTGGAAT

Table S2: Scoring sheet to determine health scores in the *P. aeruginosa* lung model

Category	Grade	Description
Warmth	0	Normal
	1	Slightly cool to touch
	3	Cool to touch
	5	Cold to touch and/or pale extremities
Fur	0	Normal
	1	Lack of grooming
	2	Slight piloerection
	3	Moderate piloerection (around head/neck)
	4	Severe piloerection (entire body)
Eyes	0	Normal
	2	Dull or squinted
	3	Discharge
	4	Sunken
Hunching	0	Normal
	2	Hunching (mild, intermittent)
	3	Hunching (moderate, persistent)
	4	Hunching (severe)
Activity	0	Normal
	1	Possible reduced activity
	2	Reduced activity
	3	Lethargic (no movement when cage moved)
	4	Lethargic, moves only when touched
	5	Non-responsive and/or persistent shaking
Breathing	0	Normal
	2	Shallow/rapid breathing (intermittent)
	3	Shallow/rapid breathing (continuous)
	4	Increased respiratory effort
	5	Gasping, extreme difficulty breathing

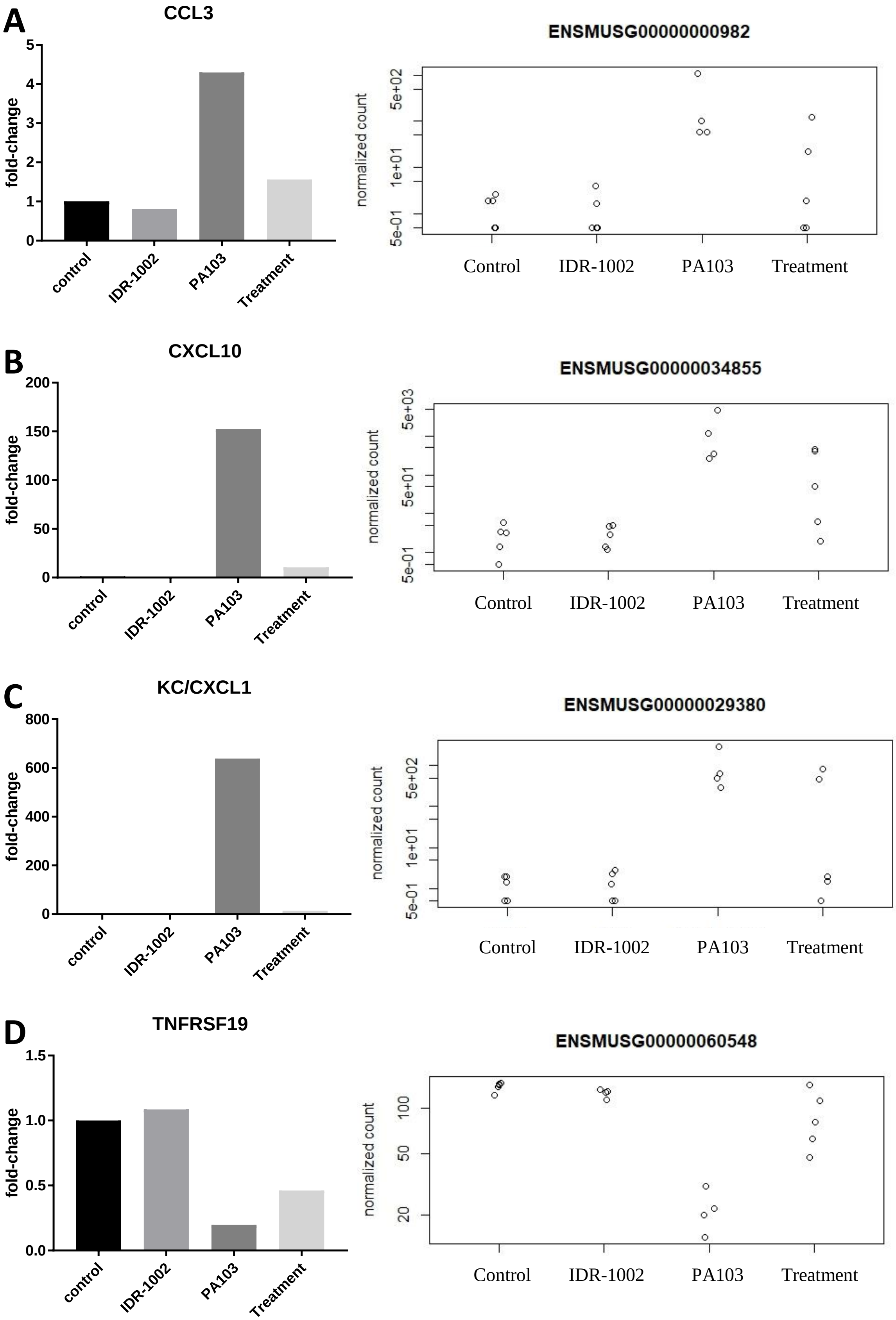
A
CCL3
fold-change
5
4
3
2
1
0
control
IDR-1002
PA103
Treatment
ENSMUSG00000000982
normalized count
5e+02
1e+01
5e-01
Control
IDR-1002
PA103
Treatment
B
CXCL10
fold-change
200
150
100
50
0
control
IDR-1002
PA103
Treatment
ENSMUSG00000034855
normalized count
5e+03
5e+01
5e-01
Control
IDR-1002
PA103
Treatment
C
KC/CXCL1
fold-change
800
600
400
200
0
control
IDR-1002
PA103
Treatment
ENSMUSG00000029380
normalized count
5e+02
1e+01
5e-01
Control
IDR-1002
PA103
Treatment
D
TNFRSF19
fold-change
1.5
1.0
0.5
0.0
control
IDR-1002
PA103
Treatment
ENSMUSG00000060548
normalized count
100
50
20
Control
IDR-1002
PA103
Treatment

Fig. S1: qPCR confirmation and comparison to RNA-Seq count data. Lung samples were collected from mice at 18 h and used for RNA-Seq and qPCR validation. The qPCR data were analyzed using the comparative Ct method and normalized to the average of two reference genes (EEF2 and B2M) and fold-change calculated relative to the control group. (A-D) Fold-changes (left) and RNA-Seq counts (right) for select genes, (A) CCL3, (B) CXCL10, (C) KC/CXCL1, (D) TNFRSF19.
